# Supplementary material for: On the identification of differentially-active transcription factors from ATAC-seq data
Source: bioRxiv. 2024 Mar 10:2024.03.06.583825. Preprint. [Version 2] doi: 10.1101/2024.03.06.583825 (PMC10942475; doi:10.1101/2024.03.06.583825)
Supplement: Supplementary file 1 [file media-1.pdf]

On the identification of differentially-active transcription fators from ATAC-seq data

Supplementary Figures

Gerbaldo, Sonder et al.

06 March, 2024

Supplementary Figure 1

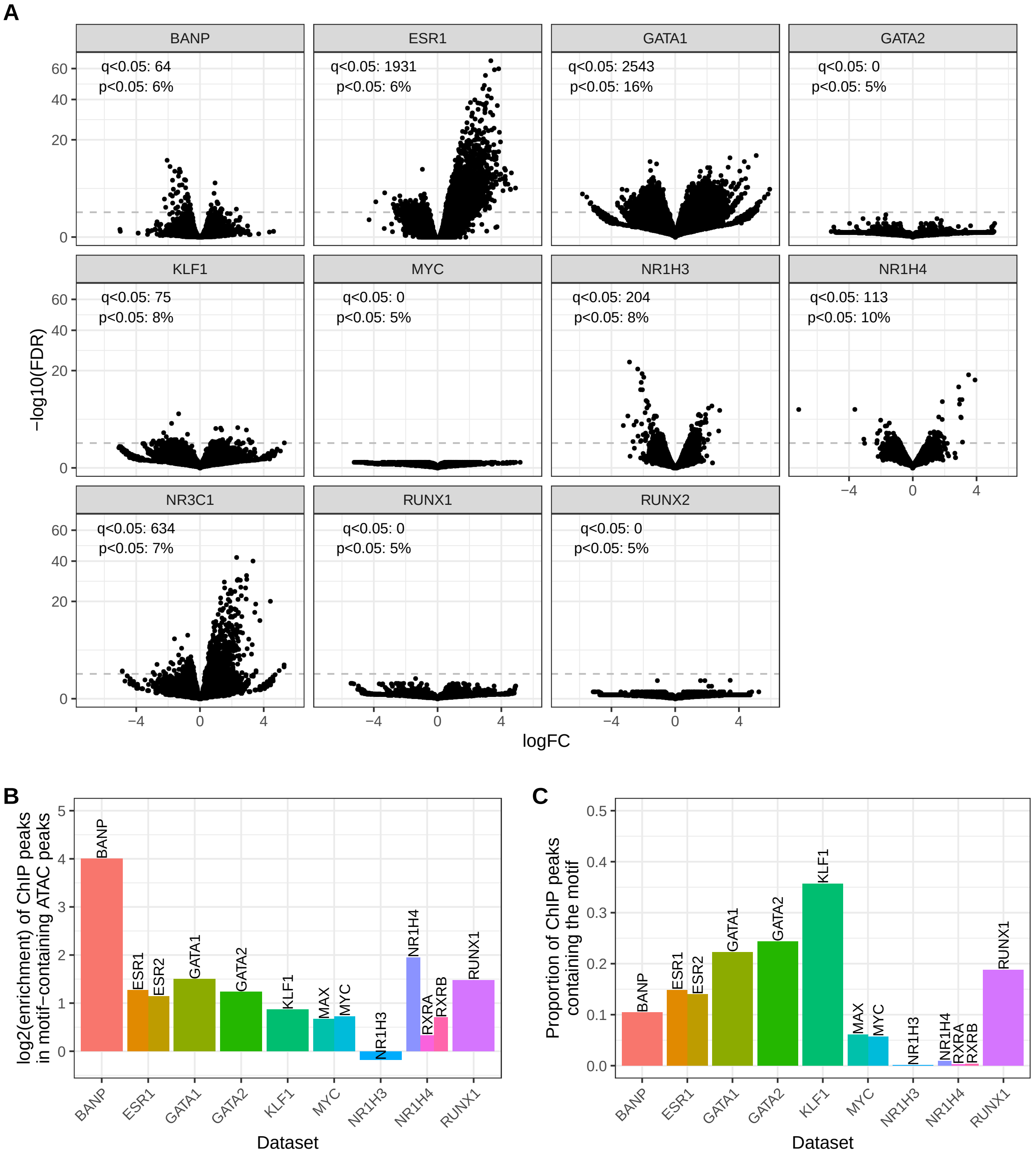

Supplementary Figure 1 : Overview of the benchmark datasets and TFs. **A:** Volcano plot of the peak-level differential accessibility analyses, illustrating the extent and significance of changes upon treatment in each dataset. The dashed line represents a 0.05 FDR threshold, and indicated are the number of ATAC peaks passing this threshold, as well as the proportion of peaks with an uncorrected p-value lower than 0.05. **B:** Enrichment for experimental binding sites of the factor (main TF after which the datasets are named) in ATAC peaks containing the respective motifs, versus all ATAC peaks. **C:** Proportion of ChIPseq peaks (overlapping ATAC peaks) that contain the respective motif.

Supplementary Figure 2

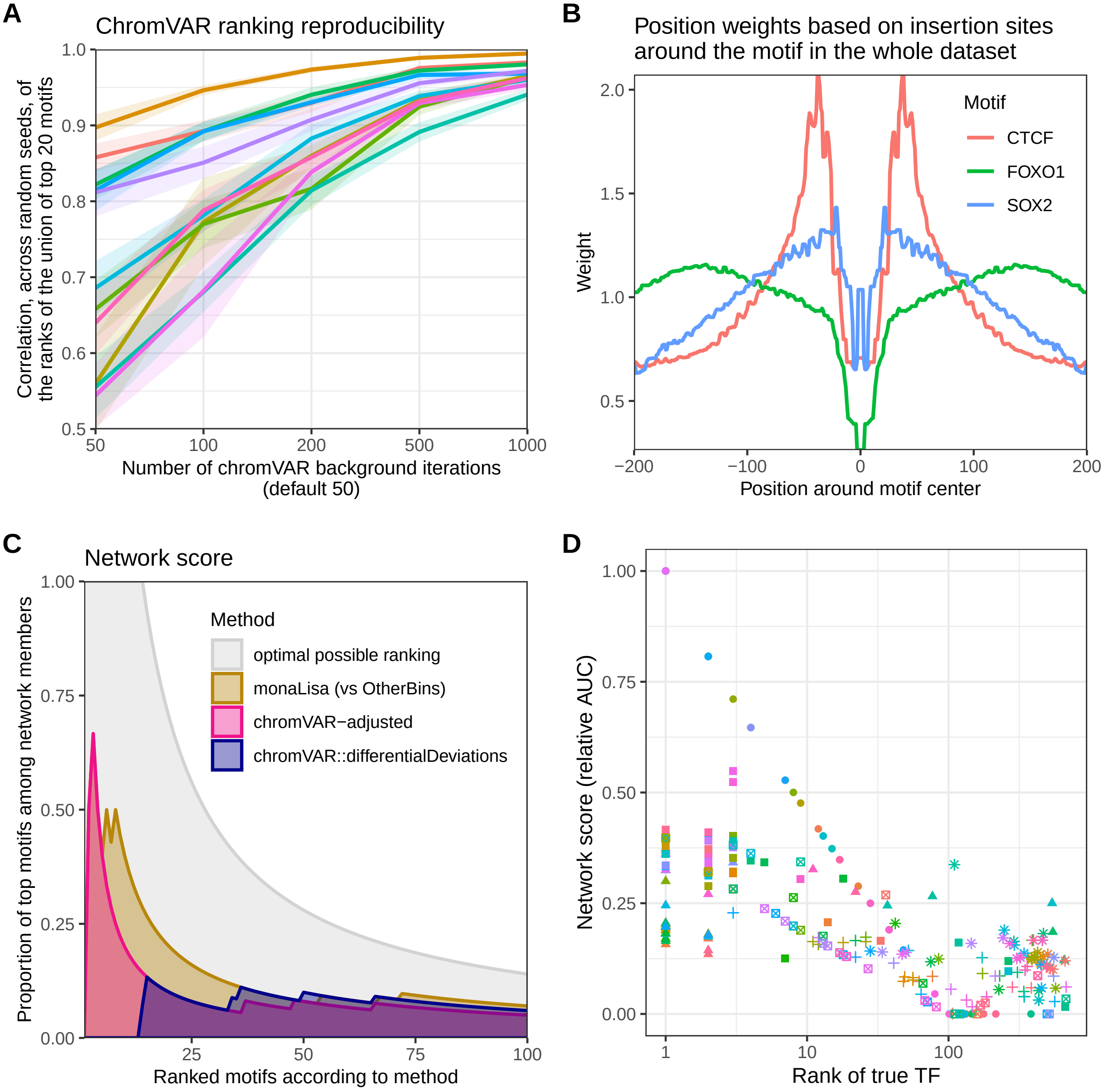

Supplementary Figure 2 A: Reproducibility of chromVAR-based differential accessibility ananylsis. Each color represents a dataset. The line and shaded area respectively represent the mean and standard error of the pairwise correlations across 7 independent runs of the chromVAR>limma pipeline for each dataset and setting, of the ranks of the (union of) top 20 motifs. **B: Illustration of the motif-specific position weights for the insertionModel method. C: Illustration of the computation of the network score.** The motifs are ranked for each method, and for each k=1:100 the proportion of top k motifs that are part of the known network of the true TF is computed. The area under the curve is then reported, relative to the AUC of the best possible motif ordering for that factor (gray area). The example plotted here is from the GATA1 dataset. **D: Agreement between the two rank-based metrics.** Each point represents the results of a method (colour) in a dataset (shape). The network score is not entirely comparable across datasets. Beside this effect, and except at very low (1-3) or high (>100) ranks (i.e. where the ranks stop being discriminatory), there is a good agreement between the two metrics.

Supplementary Figure 3

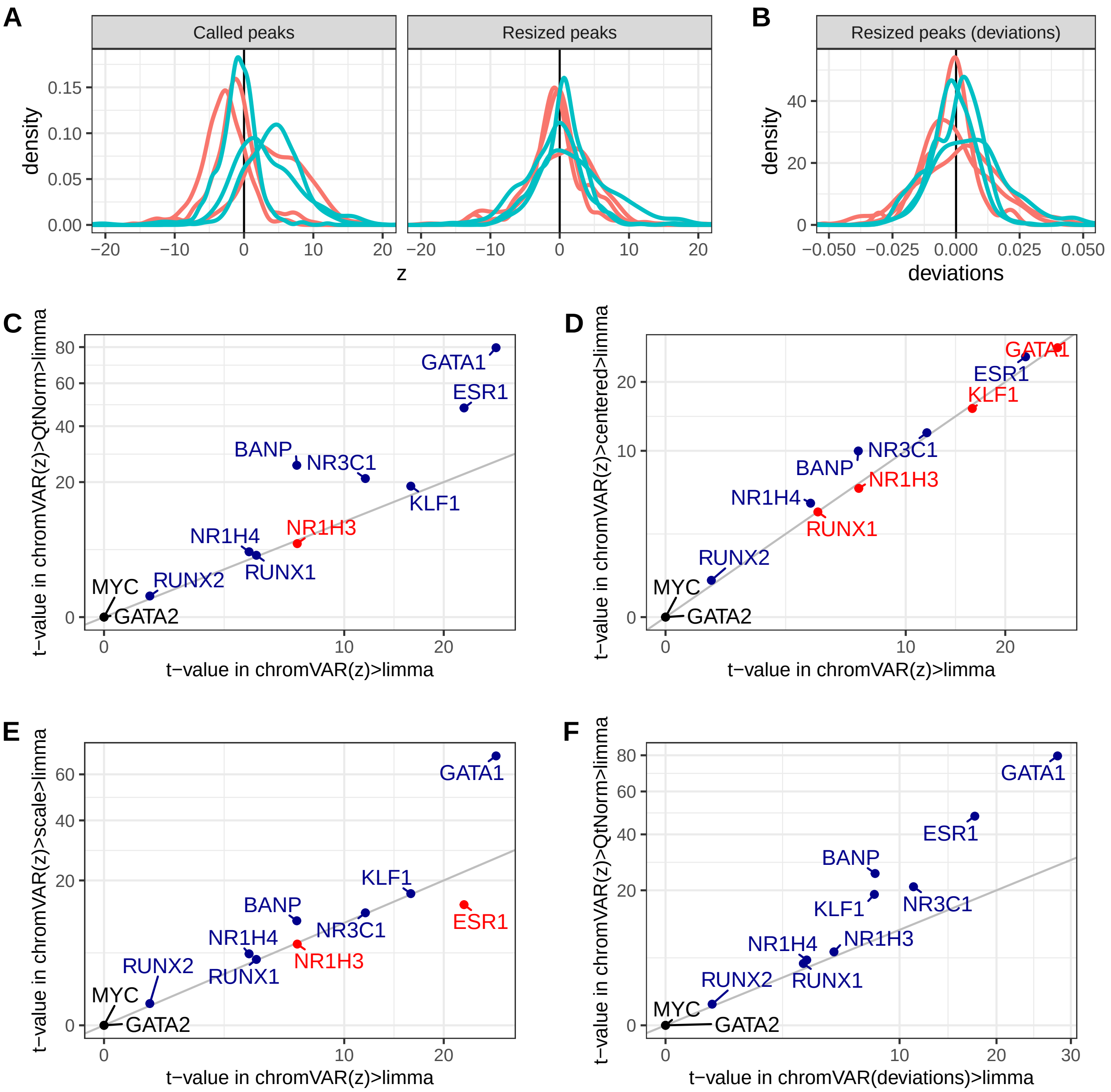

Supplementary Figure 3 : Differences in the chromVAR motif score distributions across samples, and impact of their normalization A-B: Example of a subset of ATAC samples from Caradonna, Paul and Marrocco (2022) (GSE200670), where different samples show different shapes of motif z-scores distributions. When peaks are not resized to all have the same width, as recommended, the distributions are often globally shifted (**A**, left). Even when peaks are resized (**A**, right), differences in the width of the z-scores distributions which are not related to experimental groups can persist in some datasets. This is not specific to the z-scores, but also present in the bias-corrected deviations (**B**). **C-F:** For each comparison, the limma's t-value for the true TF is reported; for datasets involving a downregulation of the TF, the sign of the t-value was inverted. For ease of visualization, t-values below 0 were set to zero, and the axes were square-root transformed. TF in blue show an improvement, while those in red show a worsening, and those in black are unchanged.

Supplementary Figure 4

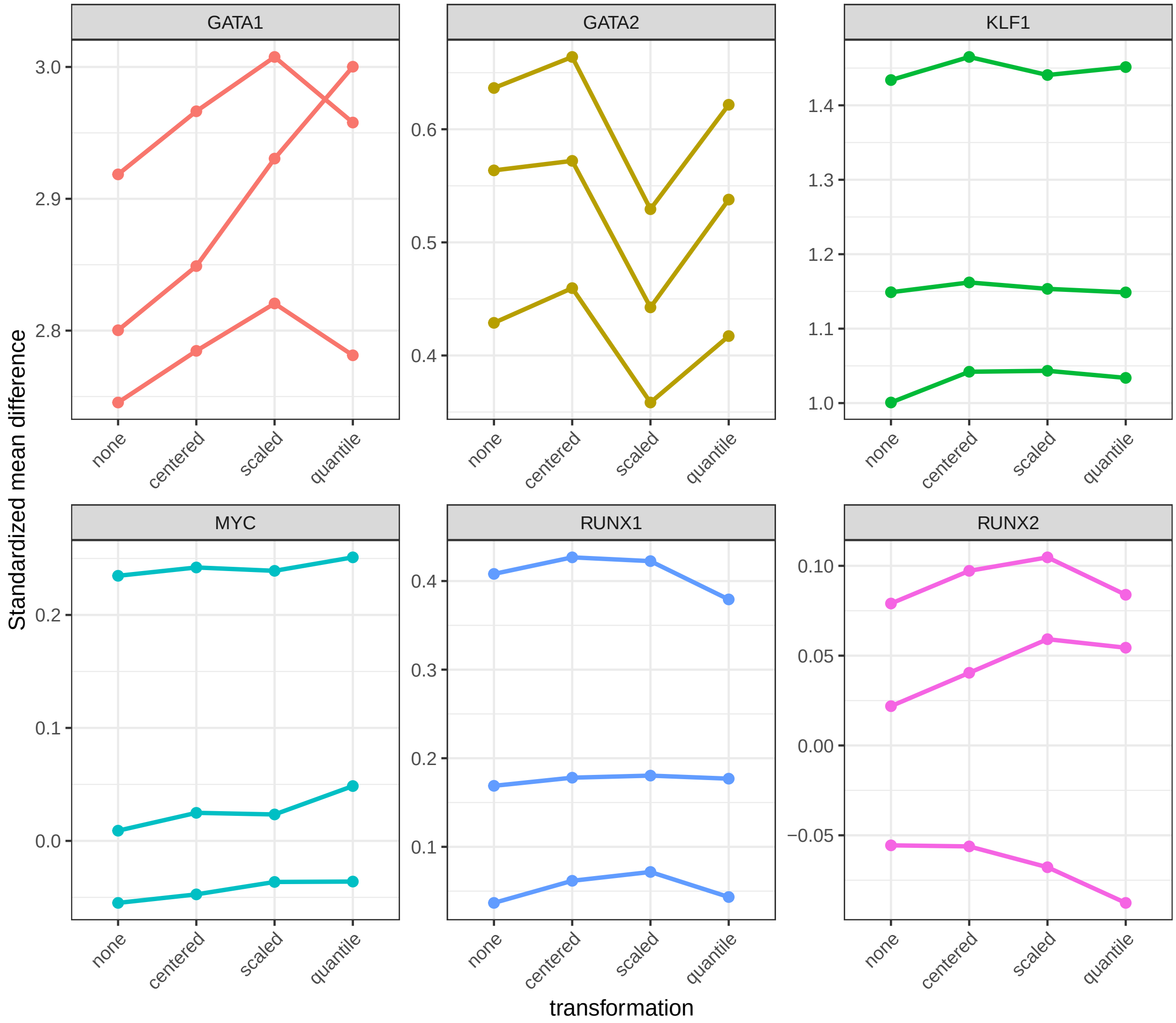

Supplementary Figure 4 : Impact of chromVAR motif z-score normalization at the single-cell level. As a proxy to signal-to-noise ratio, we computed the standardized mean difference of the respective motif for each guide RNA (individual lines) to the mean of the control guide RNAs. Centering is always beneficial (Wilcoxon p<8e-06), unit-variance scaling is often beneficial (Wilcoxon p~0.07) but sometimes detrimental, and quantile normalization also tends to be beneficial (Wilcoxon p~0.041).

Supplementary Figure 5

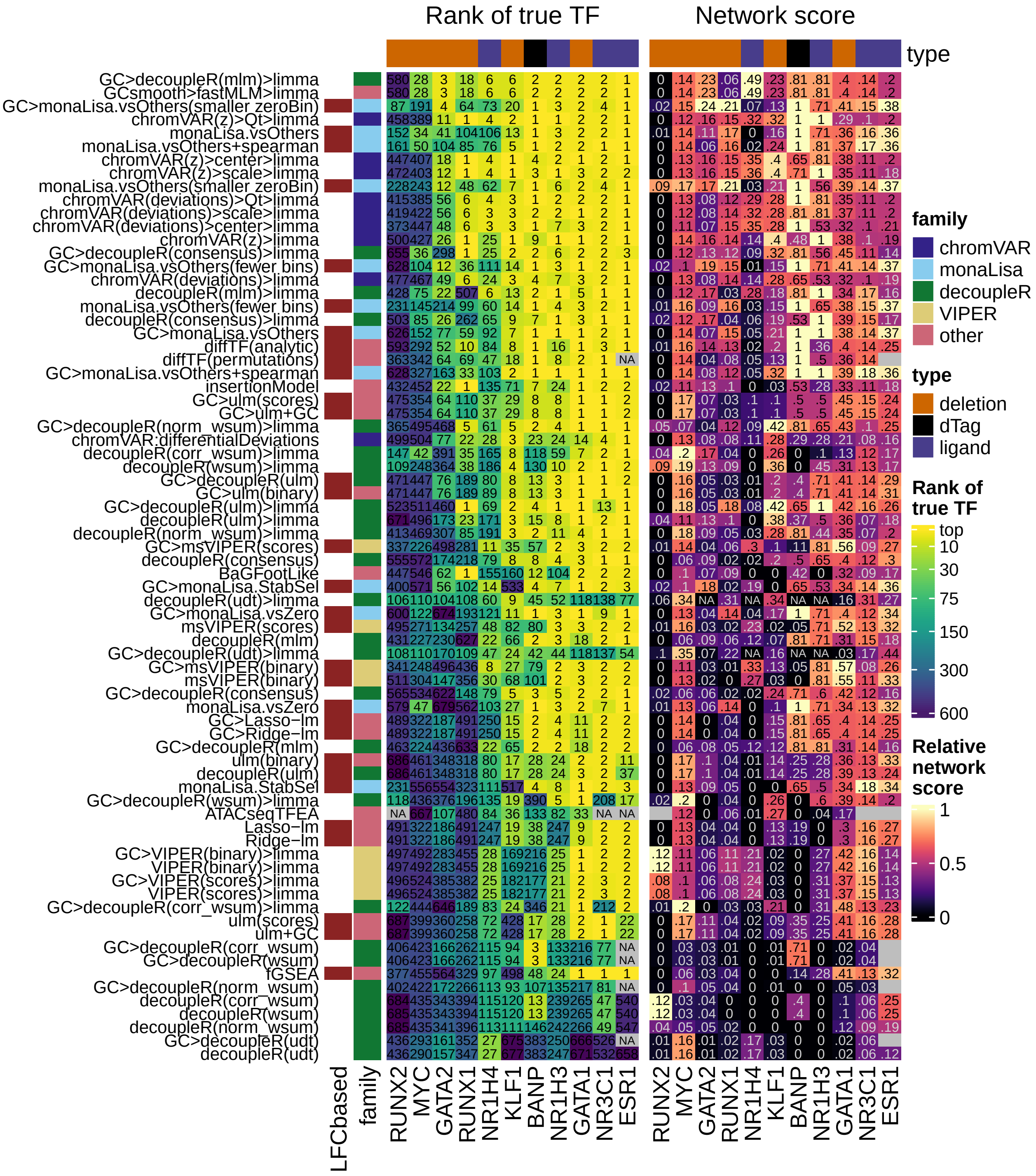

Supplementary Figure 5 : Rank-based metrics of all variants, including smooth quantile normalization in GC bins.

Supplementary Figure 6

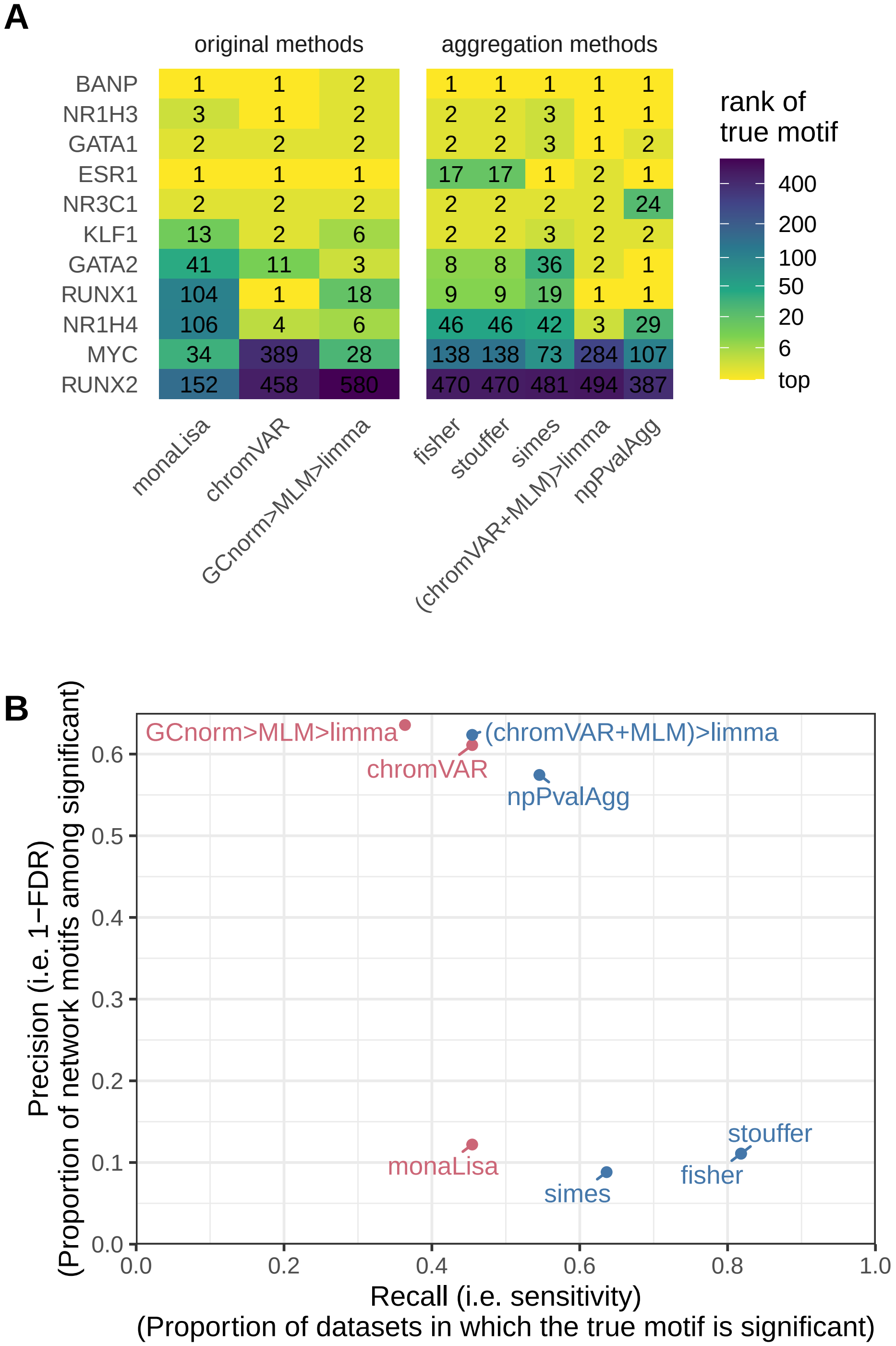

Supplementary Figure 6 : Simple aggregation of top methods' results does not improve inferences. The results of the top method from each of the two families of approach (on the left) are compared to those of simple aggregation methods (on the right). Beside the established Fisher's, Stouffer's and Simes' methods, a rank-based permutation approach was tested, establishing the probability of having a sum of ranks across methods lower or equal to the observed one.

Supplementary Figure 7

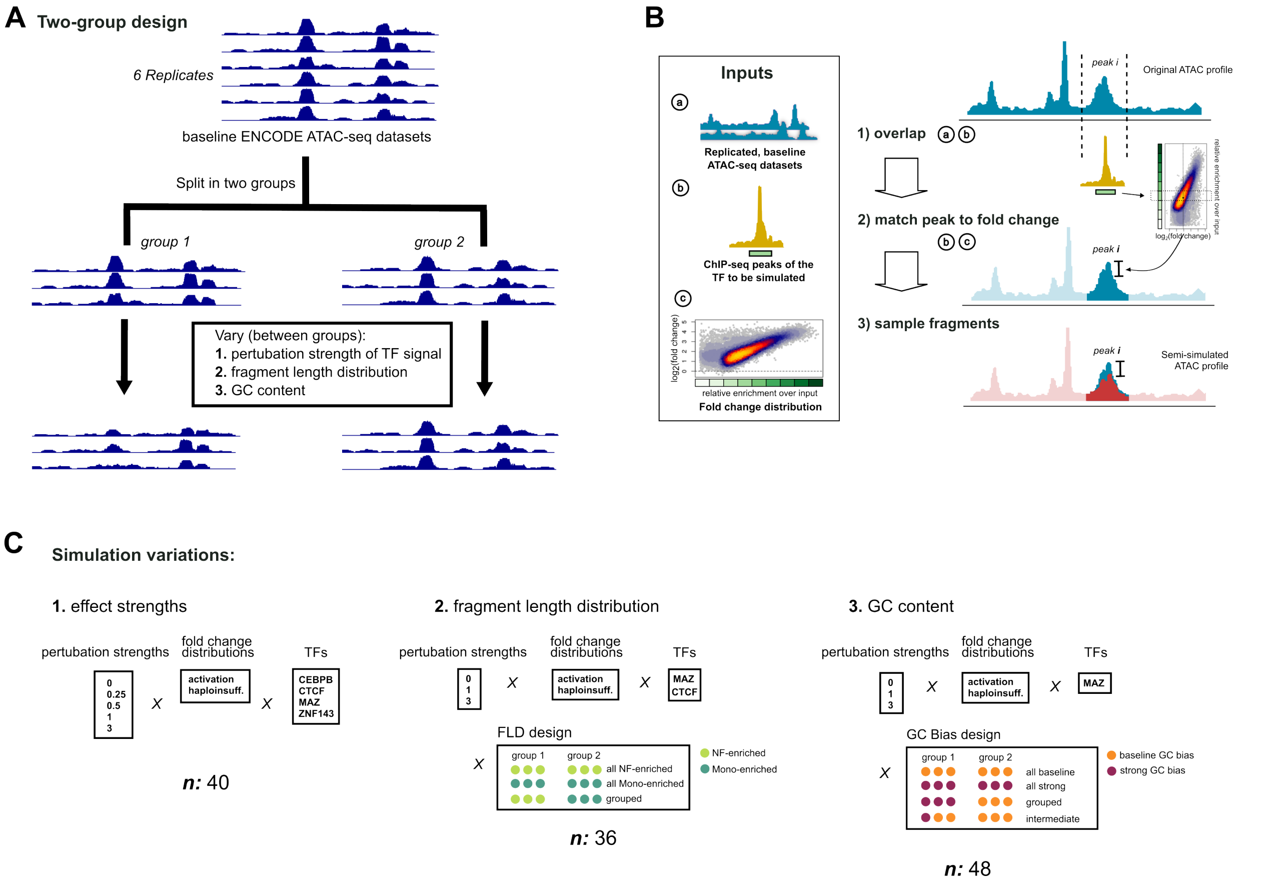

Supplementary Figure 7 : Overview of the semi-simulations. **A:** 6 baseline ATAC-seq lymphoblastoid cell lines (LCLs) are divided into two groups. Differences will be introduced through downsampling of fragments in one of the groups, and eventual per-sample GC and fragment length biases are introduced. **B:** The extent of downsampling is based on the enrichment over the input of the ChIP-seq peak, following one of two differential activity scenarios: an activation and a haploinsufficiency (see Figure 4B). The magnitude of the effect is further scaled up and down (perturbation strength). **C.** Settings used for the semi-simulated datasets. The resulting datasets can be divided into three groups, a first set where only the strength of the perturbation was varied by a factor (perturbation strength), a second and third one were additionally differences in respectively fragment length distribution and GC were introduced across samples.

Supplementary Figure 8

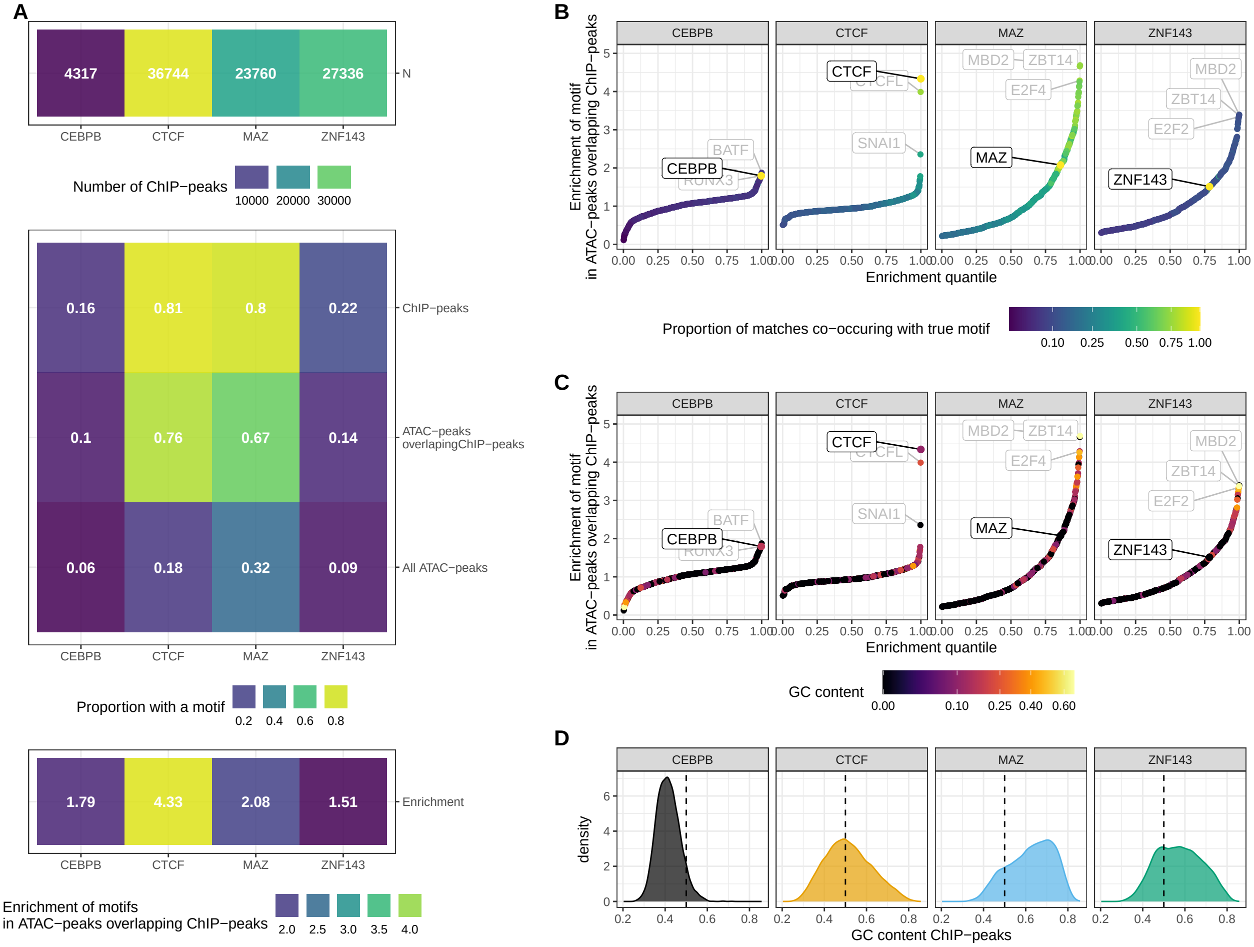

Supplementary Figure 8 : Overview of the simulated TFs. Data shown correspond to the semi-simulated datasets with a pertubation strength of 1 in the activation paradigm. **A:** Number of peaks, proportion of ChIP-seq and/or ATAC-seq peaks with a motif, and fold-enrichment of ATAC-peaks overlapping ChIP-seq peaks (i.e. bound peaks) for the motif across the four TFs. **B-C:** Enrichment of all tested motifs among the ATAC-seq peaks overlapping ChIP-seq peaks (relative to all ATAC-seq peaks), with the true motif labeled in black. The dots are colored either by co-occurence with the true motif (**B**) or by GC content (**C**). **D:** GC content distributions of the ChIP-seq peaks of each TF.

Supplementary Figure 9

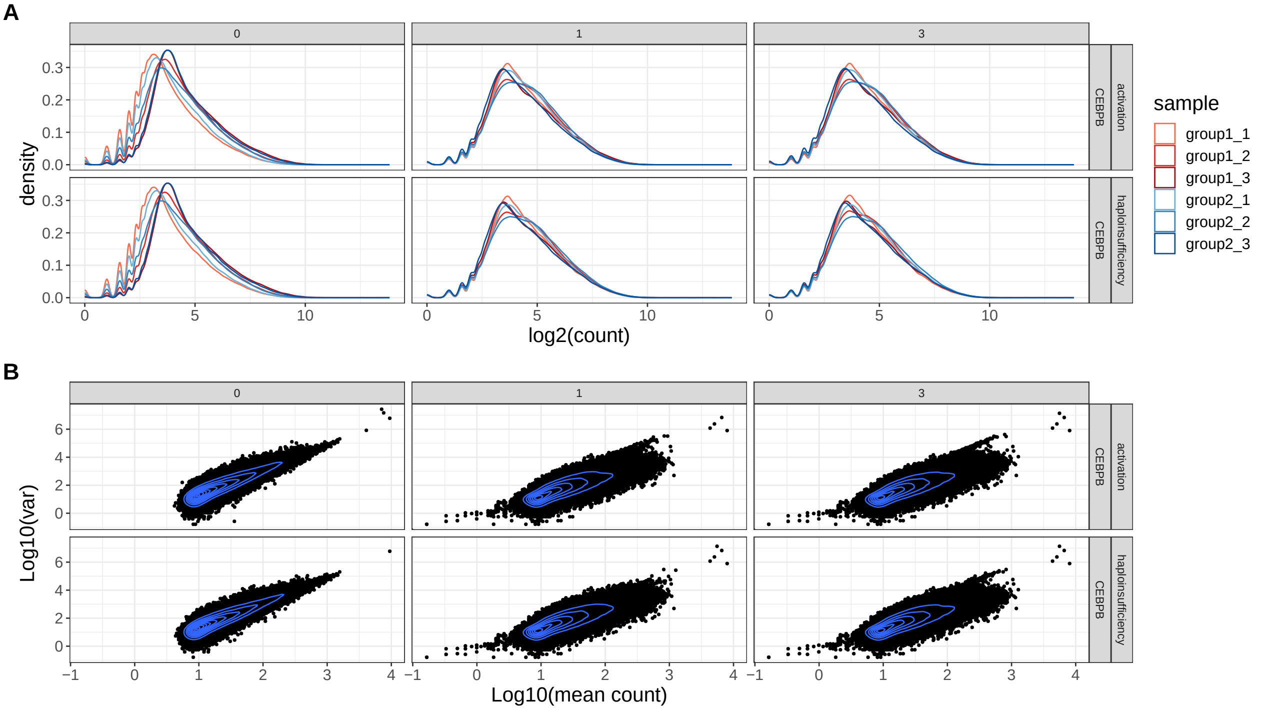

Supplementary Figure 9 : Distributions of fragment counts and variance of the simulations A: Per peak fragment count distributions of the original (denoted ”0”) and simulated samples with 1-fold and 3-fold perturbation strengths, for an example TF (CEBPB) across both paradigms. **B:** Mean-variance relationship of the original (denoted “0”) and simulated samples.

Supplementary Figure 10

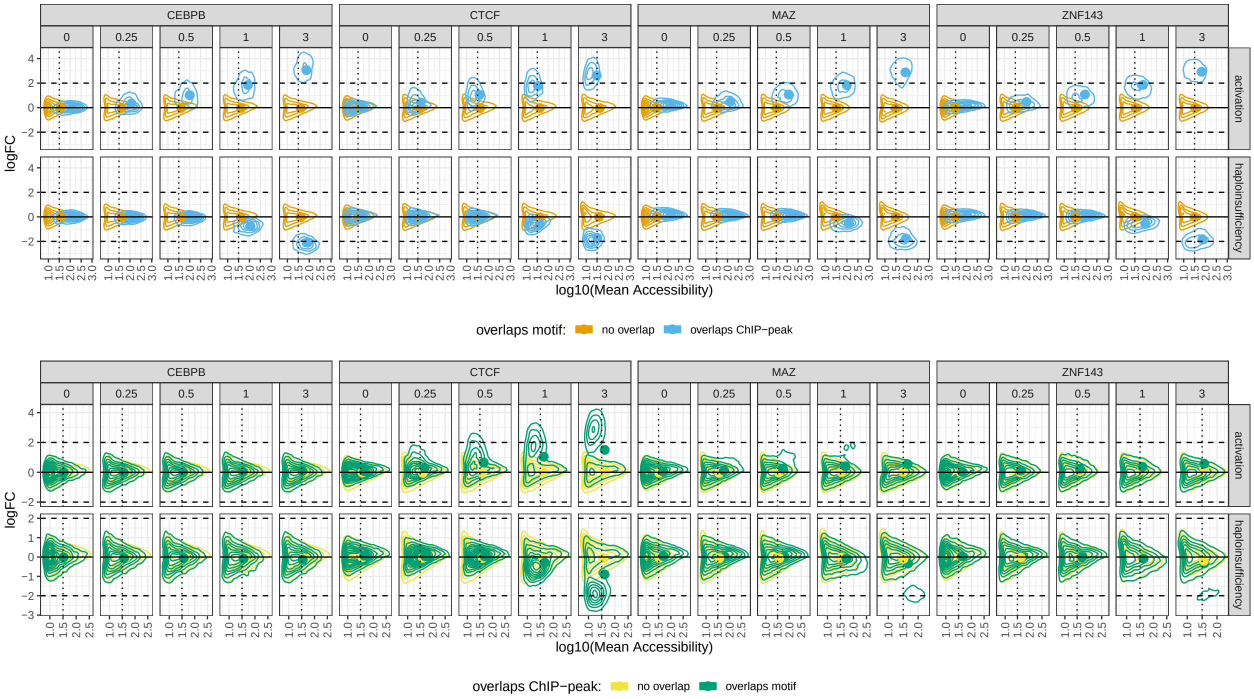

Supplementary Figure 10 : MA plots of the simulated effects For each factor, simulated paradigm and perturbation strength, the distribution of peaks log2FC of raw fragment counts between conditions as a function of mean accessibility is plotted as 2d kernel density, separating either (**A**) ATAC-seq peaks overlapping a ChIP-seq peak or not, or (**B**) ATAC-seq peaks containing a motif or not for the TF. For visibility, the peaks were subsampled to a more comparable size across the simulated datasets (10ˆ5 peaks per dataset). Colored Points indicate the means of the respective distributions.

Supplementary Figure 11

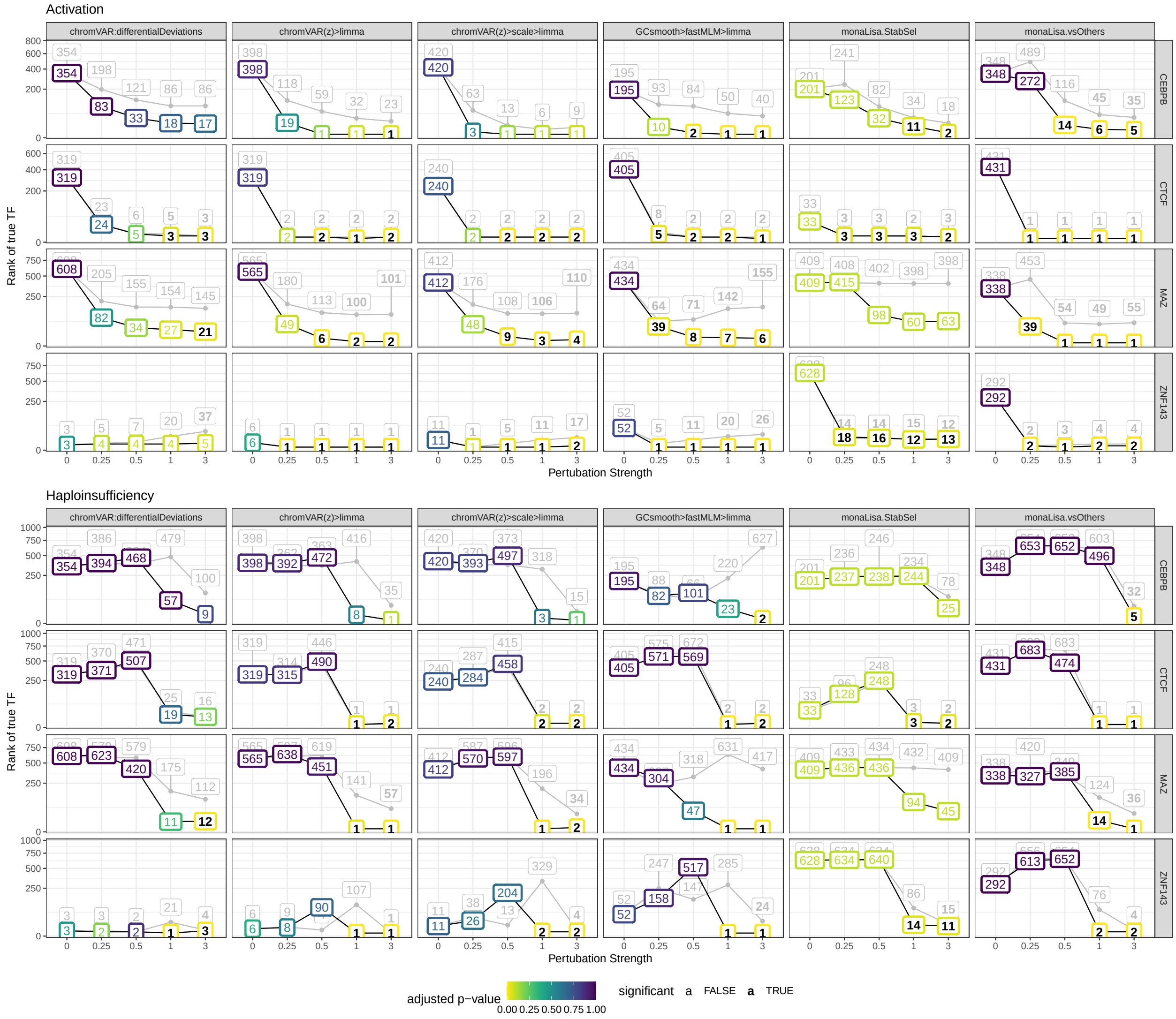

Supplementary Figure 11 : Positive control of the simulations Shown are ranks and significance assigned by each method to the true motif across simulation parameters (TF, paradigm and perturbation strength). In light gray are the normal simulations (as shown in main Fig 4), while dark line and colored boxes correspond to the positive controls where a difference between the groups was only introduced in the peaks overlapping a motif of the true TF.

Supplementary Figure 12

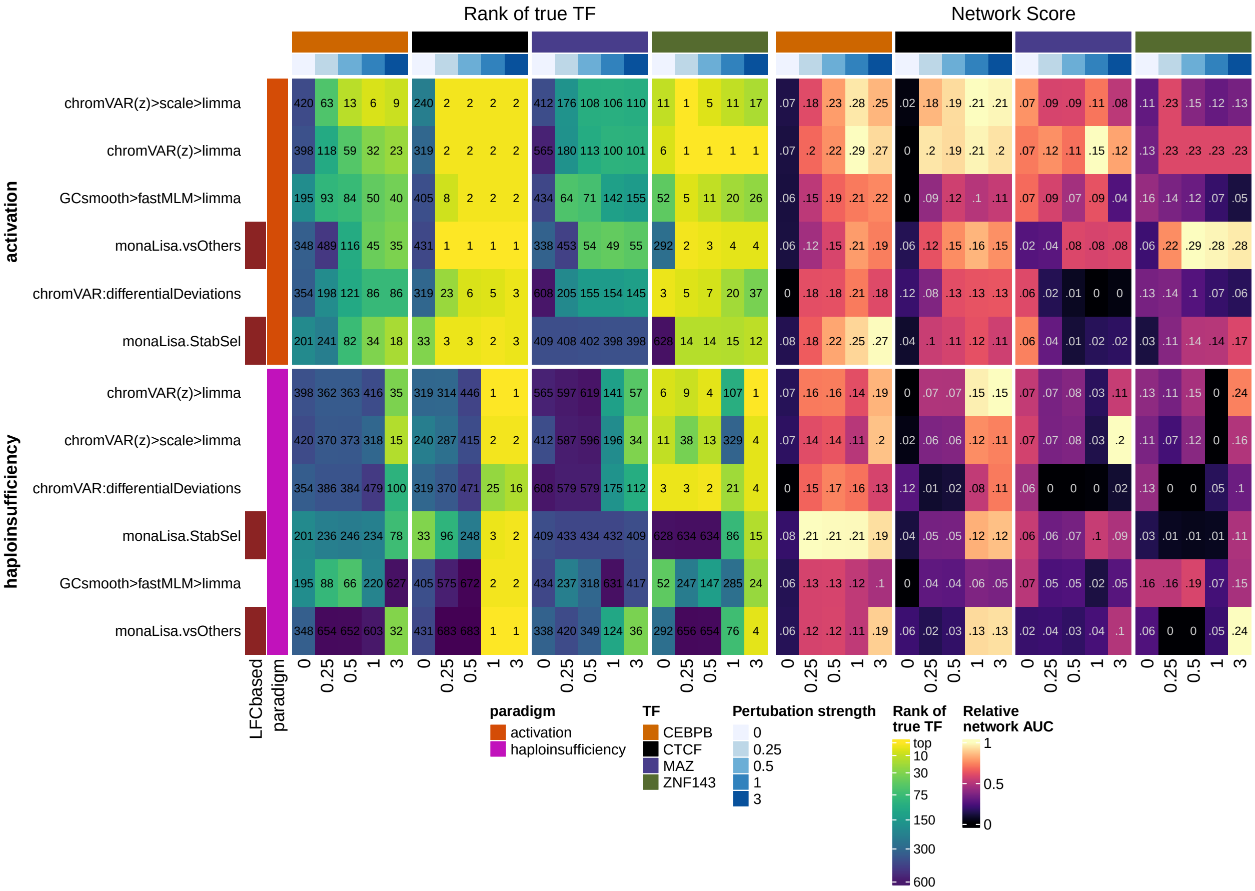

Supplementary Figure 12 : Heatmap of performance of variants of top methods on the semi-simulated datasets. As in main Fig 1, the left part of the heatmap shows the ranks, while the right part shows the corresponding network scores obtained on the simulated datasets.

Supplementary Figure 13

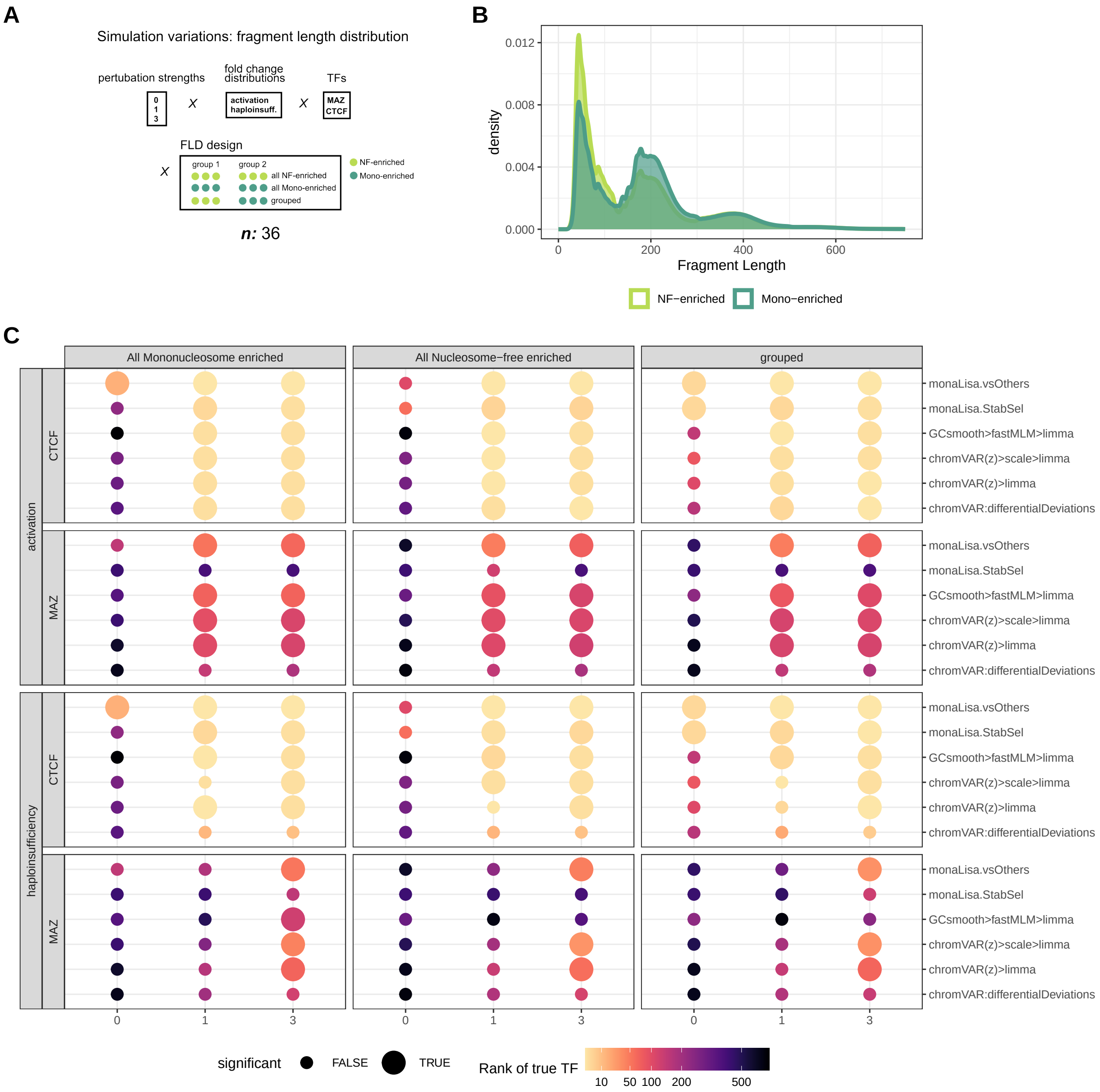

Supplementary Figure 13 : Robustness to simulated variations in fragment length. In addition to the simulated TF effect, two different fragment length distributions (**B**) were simulated by differential sampling of the fragments according to their length for the different samples as in scheme **A**. The distribution in light green corresponds to sampling mononucleosome containing fragments with higher probability, whereas for the distribution in dark green nucleosome-free fragments were sampled preferentially. The results of the methods are shown in **C**. Colors of the points correspond to the rank of the true TF obtained, point size if the corresponding adjusted p-value was found to be significant (adjusted p-value <= 0.05).

Supplementary Figure 14

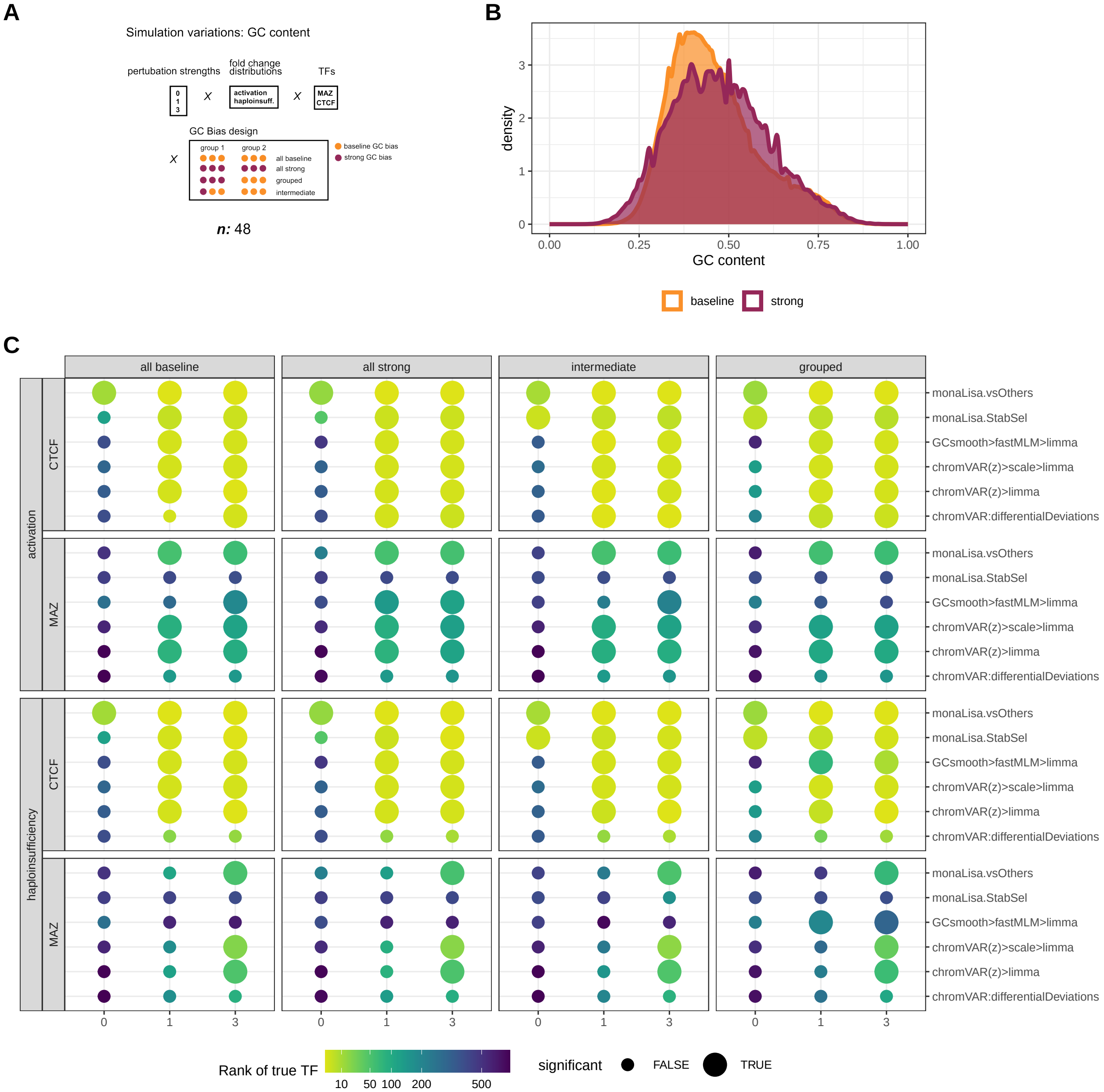

Supplementary Figure 14 : Robustness to simulated variations in GC content. In addition to the simulated TF effect, two different GC content distributions (**B**) were simulated by differential sampling of the fragments according to their GC content for the different samples as in scheme **A**. The results of the methods are shown in **C**. Colors of the points correspond to the rank of the true TF obtained, point size if the corresponding adjusted p-value was found to be significant (adjusted p-value <= 0.05).

Supplementary Figure 15

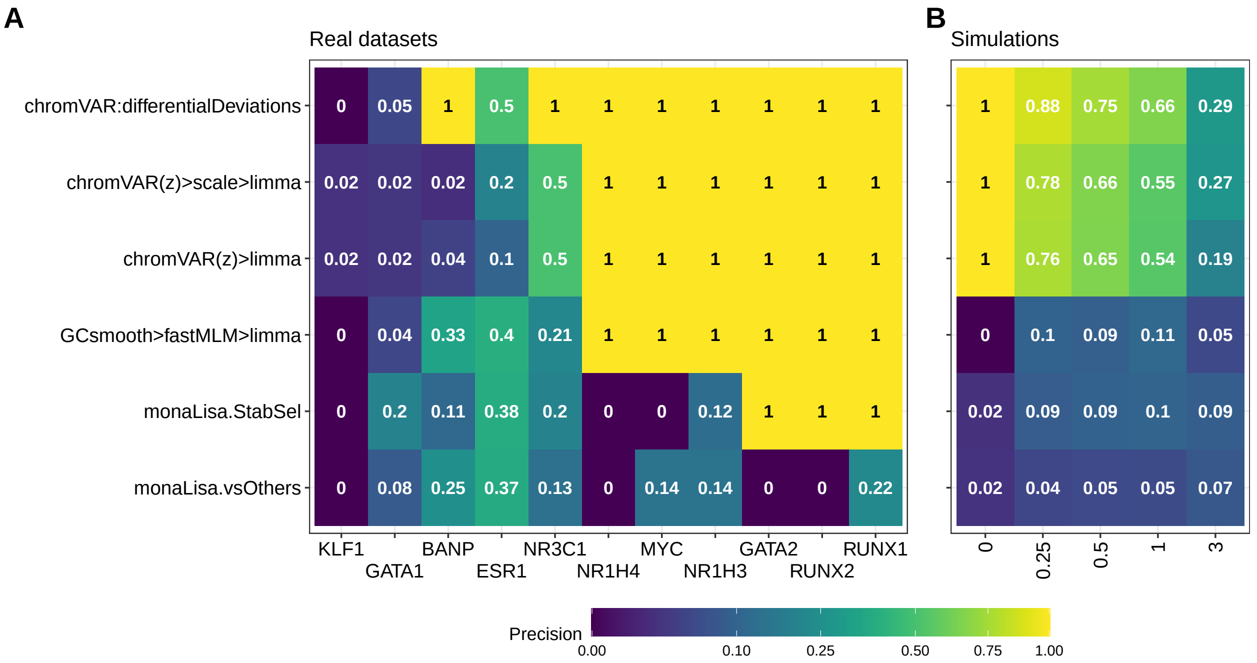

Supplementary Figure 15 : Estimated precisions per dataset Heatmap of precision estimates obtained per dataset and method on the 11 real datasets (**A**), as well as averaged across each perturbation strength of the simulations (**B**).

Supplementary Figure 16

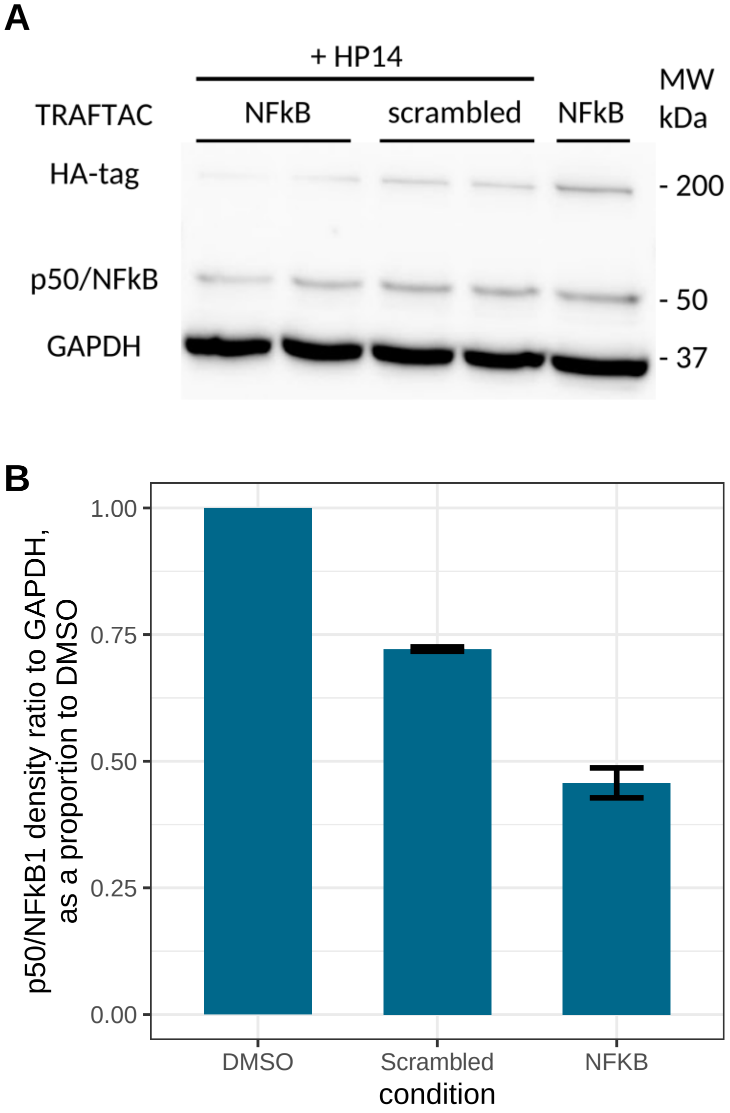

Supplementary Figure 16 : Western blot confirms the downregulation of p50/NfKB. Western blot (**A**) and corresponding densitometry showing a downregulation of p50/NFkB in HEK cells treated with NFkB TRAFTACs versus scrambled TRAFTACs.
